# Supplementary material for: Combined Presence in Heterozygosis of Two Variant Usher Syndrome Genes in Two Siblings Affected by Isolated Profound Age-Related Hearing Loss
Source: Biomedicines. 2023 Sep 28;11(10):2657. doi: 10.3390/biomedicines11102657 (PMC10604119; doi:10.3390/biomedicines11102657)
Supplement: Supplementary file 1 [file biomedicines-11-02657-s001.zip › biomedicines-2595912-supplementary.pdf]

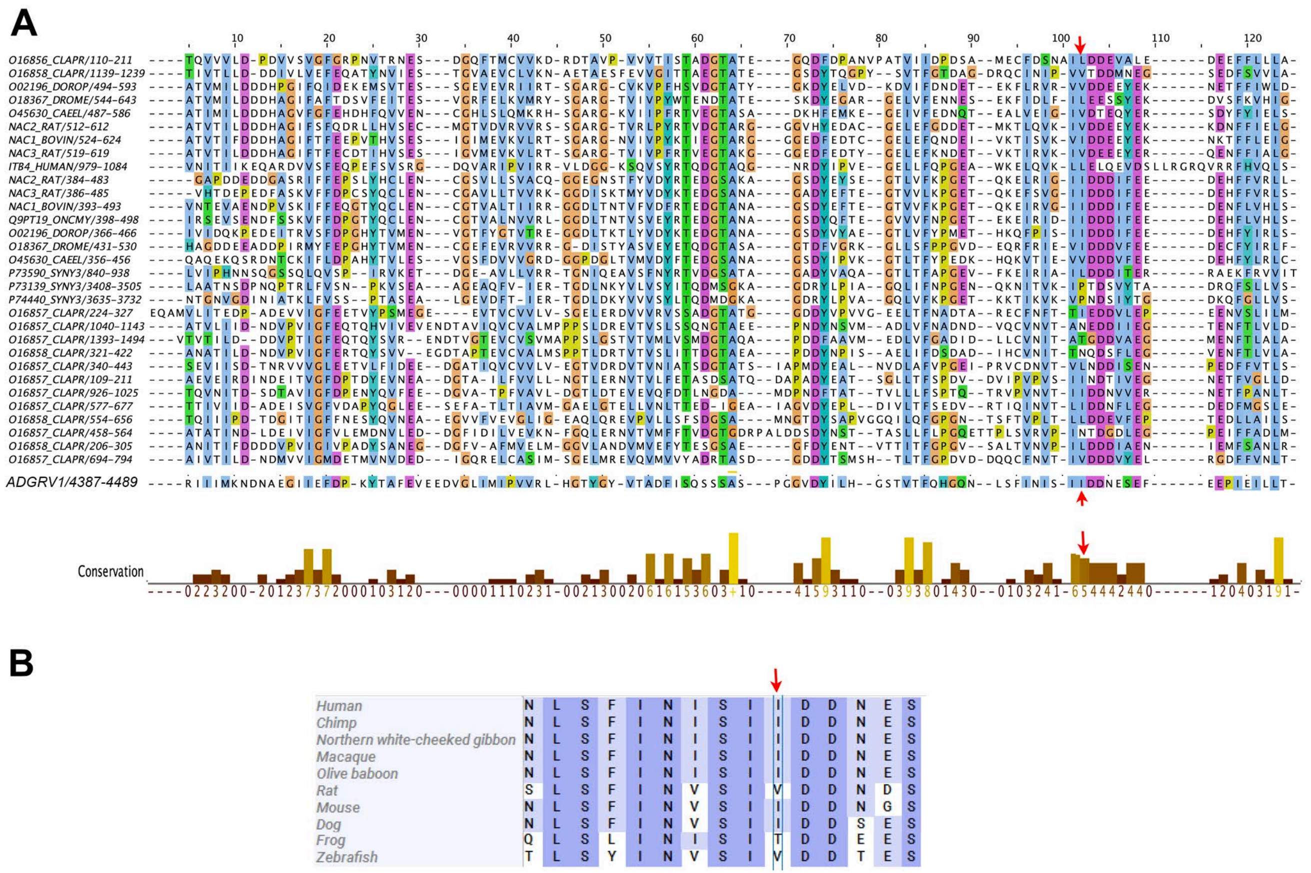

Fig. S1. A: Seed alignment of Calx β domain (PF03160) in PFAM database together with the Calx-β 30 domain of ADGRV1 (shown in the last line of the alignment). Residue coloring is according to the Clustal X Color Scheme (https://www.jalview.org/help/html/colourSchemes/clustal.html). The conservation plot (Livingstone and Barton Comput Appl Biosci 6:745, 1993) below the alignment was generated using the Jalview analysis package (Waterhouse et al. Bioinformatics 25:1189, 2009). The shading of the bars from brown to yellow reflects the conservation number indicated below the bars. The position of the mutation is indicated by the vertical red arrows. Note that this position is predominantly occupied by hydrophobic residues. B: MSA of the region surrounding I4473)red arrow) in the 30th Calx-β domain of vertebrate orthologs selected with Alamut Visual Plus. The residue is moderately conserved among orthologs and the position is occupied by a hydrophobic residue in all but one case.

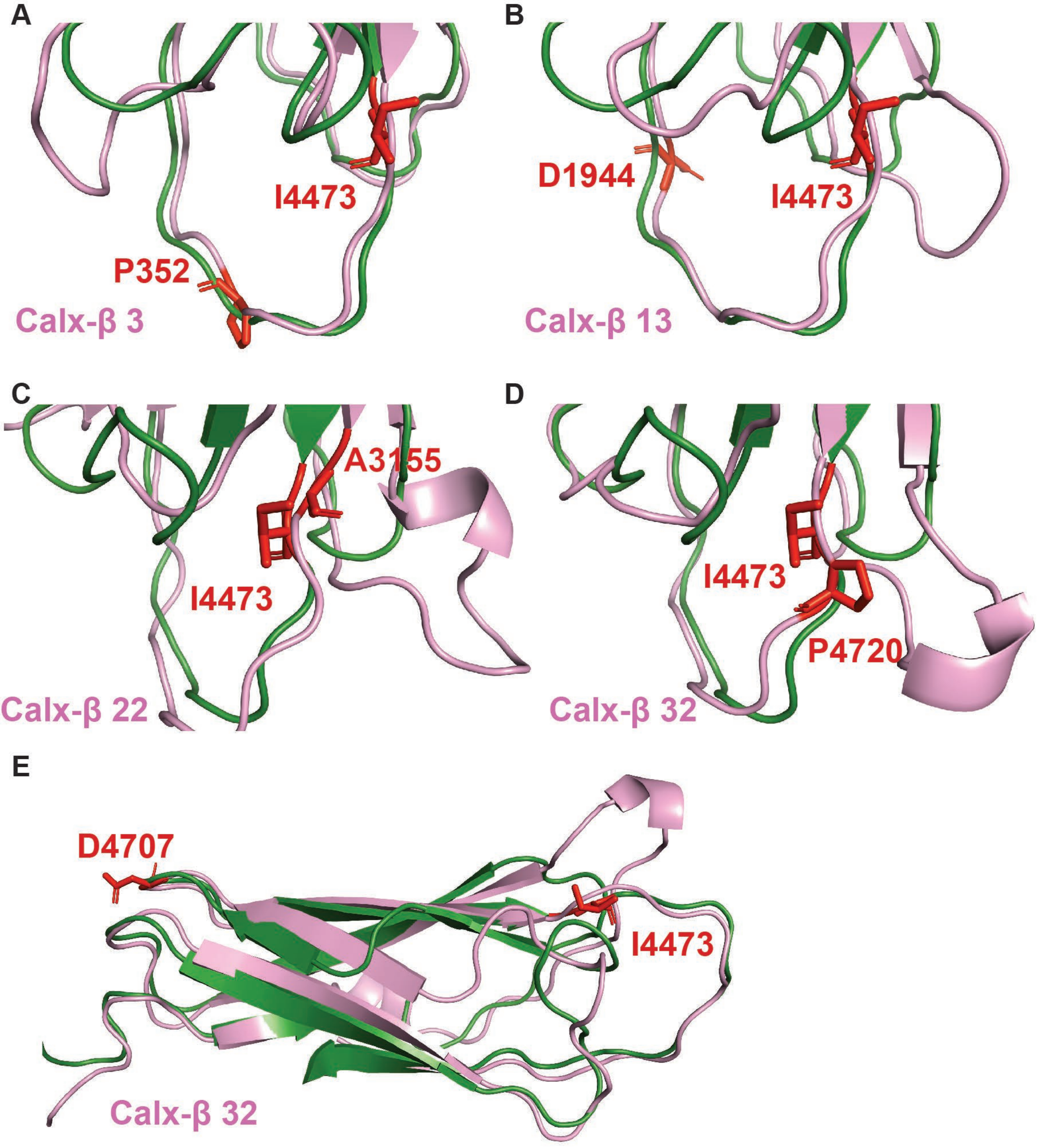

Fig S2: Positions of the variant residues analyzed in Table 1, according to ColabFold predicted structures of each Calx- β domain. In all panels, the Calx-β domain 30, which harbors the I4473N variant, is shown in green and aligned to the other analyzed variant-carrying Calx-β domain shown in pink. The number of each Calx-β domain and the considered variant residue are indicated in each panel.
